# New teenage challenge: vaping and pregnancy

**DOI:** 10.3389/fmed.2026.1940178

**Published:** 2026-08-25

**Authors:** Mohamed N. Ahmed, Tony Eczema, Nahla Zaghloul

**Affiliations:** 1 Department of Pediatrics, Division of Neonatology, University of Florida, Gainesville, FL, United States; 2 Department of Medicine, Division of Pulmonary Medicine, Stony Brook University, Stony Brook, NY, United States

**Keywords:** electronic cigarettes (e-cigarettes, ECs), maternal smoking, nicotine, perinatal outcomes, preterm birth, small for gestational age, teenage pregnancy, vaping

## Abstract

The use of nicotine and tobacco products in any form is unsafe, and nearly all tobacco product initiation occurs during adolescence. Vaping via electronic cigarettes (ECs) among young individuals remains a major public health concern. According to the 2025 National Youth Tobacco Survey, 5.2% of U.S. middle and high school students currently use ECs (7.1% of high school students and 2.6% of middle school students), making ECs the most consumed tobacco product among youth. While combustible cigarettes deliver high concentrations of carbon monoxide and combustion byproducts, EC aerosol contains variable levels of nicotine alongside chemical toxicants such as formaldehyde, acetaldehyde, nickel, and lead. Systemic nicotine delivery from ECs varies considerably by device design, liquid formulation, power output, and vaping behavior, but can equal or exceed that of conventional cigarettes. Cumulative clinical and epidemiological evidence indicates that exclusive EC use and dual use with combustible cigarettes during pregnancy are associated with adverse perinatal outcomes, including preterm birth and reduced birth weight. This perspective highlights epidemiology, fetal toxicology, adolescent susceptibility, and exposure-specific perinatal outcomes.

## Background

Electronic cigarettes (ECs) are battery-powered devices that use a heating element to vaporize an e-liquid solution into an inhalable aerosol. E-liquids typically comprise propylene glycol, vegetable glycerin, distilled water, flavorings, and variable concentrations of nicotine or other active compounds. Current device architectures fall into three primary categories: (1) disposable single-use systems, (2) user-refillable tank or mod systems, and (3) cartridge or pod-based devices utilizing pre-filled e-liquid pods.

Systemic nicotine yield and toxicant exposure from ECs are not uniform; they vary markedly depending on device wattage, coil resistance, e-liquid formulation (e.g., freebase nicotine vs. protonated nicotine salts), and user vaping topography (puff duration, volume, and frequency) (1, 2). Under operational conditions, modern high-power devices and nicotine-salt formulations can deliver blood nicotine concentrations comparable to or higher than those produced by combustible tobacco cigarettes (2).

## Epidemiology

Tobacco product use in any form during adolescence poses established health risks, including rapid development of nicotine dependence and potential transition to combustible tobacco products (1, 3). Since 2014, ECs have consistently represented the most widely used tobacco product among U.S. adolescents (4, 5).

According to the 2025 National Youth Tobacco Survey (NYTS), an estimated 9.5% of high school students and 4.1% of middle school students reported current (past-30-day) use of any tobacco product (5). Specifically regarding e-cigarettes, 5.2% of all middle and high school students (7.1% of high school students and 2.6% of middle school students) reported current past-30-day EC use (5). While national, state, and local public health policies have contributed to overall declines in youth vaping in recent years, adolescent EC consumption remains substantial (5).

Maternal smoking during pregnancy continues to affect maternal and infant health. In the United States, 2021 natality data revealed that 4.5% of pregnant individuals reported smoking combustible cigarettes during pregnancy, with the highest prevalence observed among young adults aged 20–24 years (5.8%) and 25–29 years (5.1%), followed by adolescents aged 15–19 years (4.3%) (6). Among pregnant adolescents specifically, data from the Pregnancy Risk Assessment Monitoring System (PRAMS) regarding late-pregnancy substance use indicated that 4.1% engaged in exclusive EC use, 3.2% in exclusive combustible cigarette smoking, and 1.1% reported dual use (7).

## Smoking, vaping, and pregnancy outcomes

Combustible cigarettes produce smoke containing nicotine, carbon monoxide, heavy metals, and thousands of volatile compounds that induce fetal hypoxia, placental insufficiency, and fetal growth restriction (8, 9). Because EC aerosols lack combustion byproducts like carbon monoxide, some pregnant individuals incorrectly view vaping as a safe alternative or utilize ECs for smoking cessation (10, 11). However, EC aerosols contain nicotine alongside trace or quantifiable levels of cytotoxic chemicals, including toxic aldehydes (formaldehyde, acrolein, acetaldehyde) and heavy metals (nickel, lead, chromium) derived from heating coils (12).

## Perinatal health impacts by exposure category

Evaluating the health impacts of prenatal EC exposure requires strict distinction between exclusive e-cigarette use, exclusive combustible cigarette smoking, and dual use, as their risk profiles differ significantly.

A comprehensive systematic review and network meta-analysis by Sukhato et al. provided critical clarification regarding these distinct exposure categories (13):

- **Exclusive EC Use vs. Non-Use:** Associated with a significantly increased risk of preterm birth (pooled OR 1.67, 95% CI: 1.11–2.51), but was not significantly associated with small-for-gestational-age (SGA) birth (pooled OR 1.36, 95% CI: 0.96–1.93). Effects on birth weight were analysis-dependent: pairwise meta-analysis revealed a statistically significant but modest mean birth weight reduction of −57 g (95% CI: −105 to −9 g), whereas network meta-analysis demonstrated a non-significant mean difference of −37.74 g (95% CI: −111.65 to 36.17 g) (13).
- **Exclusive Combustible Cigarette Use vs. Non-Use:** Consistently linked with robustly established adverse outcomes, including significant reductions in birth weight (−150 to −250 g), elevated risk of SGA (OR ~1.8–3.1), and increased risk of preterm birth (8, 13–15).
- **Dual Use vs. Non-Use:** Produced the broadest and most pronounced adverse associations, including significantly elevated odds of SGA birth (OR 2.55, 95% CI: 1.81–3.59), preterm birth (OR 1.63, 95% CI: 1.20–2.21), and substantial deficits in mean birth weight (13).

Focusing specifically on pregnant adolescents, an analysis of nationwide PRAMS data by Wen et al. demonstrated important age-specific nuances: exclusive combustible cigarette smoking during adolescent pregnancy was significantly associated with increased odds of SGA birth (aOR 2.51, 95% CI: 1.79–3.52) (16). Conversely, exclusive EC use (aOR 1.68, 95% CI: 0.89–3.18) and dual use (aOR 1.68, 95% CI: 0.79–3.53) were not significantly associated with SGA in this pregnant adolescent cohort (16).

## Adolescent vulnerability and developmental effects

Adolescent pregnancy presents distinct biological, neurodevelopmental, and psychological considerations. Respiratory development continues through early adulthood; inhaling heated aerosols containing fine particulates and organic chemicals can disrupt pulmonary maturation and increase long-term susceptibility to bronchial hyperreactivity (17). Furthermore, the prefrontal cortex—which governs executive function, impulse control, and decision-making—undergoes extensive synaptic pruning until the mid-20s. Nicotine exposure during this sensitive window overstimulates nicotinic acetylcholine receptors, altering neurodevelopmental architecture and augmenting vulnerability to addiction, mood dysregulation, and cognitive impairments (18, 19).

In animal models evaluating prenatal respiratory impact, Orzabal et al. demonstrated that in a mouse model, prenatal EC aerosol exposure altered fetal lung gene expression, neonatal lung morphology, and respiratory mechanics through postnatal day 10; whether these findings predict later asthma or COPD or translate to humans remains unknown (20).

Perinatal nicotine exposure also impacts central nervous system development, impairing brain cell development, neural circuitry, and neurobehavioral self-regulation in offspring, which translates clinically to increased risks of neurodevelopmental delays and attention deficits (21, 22).

## Summary comparison of perinatal outcomes

Table 1 summarizes the current evidence status for key maternal and fetal health outcomes categorized by exclusive EC use, exclusive cigarette use, and dual use relative to non-use.

**Table 1:** Perinatal outcomes by prenatal tobacco and vaping exposure pattern.

Outcome	Exclusive EC use	Exclusive cigarette use	Dual use (EC+ cigarettes)
**Preterm birth**	**Association detected** OR 1.67 (95% CI: 1.11–2.51) (13)	**Well-established harm** OR 1.2–1.5 (8, 13)	**Association detected** OR 1.63 (95% CI: 1.20–2.21) (13)
**Small for gestational age (SGA)**	**Not statistically significant/Inconclusive** Adults: OR 1.36 (95% CI: 0.96–1.93) (13) Adolescents: aOR 1.18 (95% CI: 0.52–2.68) (16)	**Well-established harm** Adults: OR ~1.8–3.1 (14, 15) Adolescents: aOR 1.48 (95% CI: 1.05–2.08) (16)	**Association detected** Adults: OR 2.55 (95% CI: 1.81–3.59) (13) Adolescents: aOR 1.31 (95% CI: 0.61–2.80) (16)
**Low birth weight/mean birth weight deficit**	**Inconclusive/Modest reduction** Pairwise: −57 g (95% CI: −105 to −9 g) Network: −37.74 g (95% CI −111.65 to 36.17) (13)	**Well–established harm** Significant deficit (−150 to −250 g) (8, 13, 15)	**Association detected** Substantial birth weight deficit (13)

## Future directions and conclusions

Understanding the precise maternal and fetal consequences of prenatal EC exposure requires careful differentiation between exclusive vaping, combustible smoking, and dual use. Current epidemiological evidence, derived primarily from observational cohorts and database registries (e.g., PRAMS), indicates that while exclusive EC use is associated with lower exposure to toxic combustion products such as carbon monoxide, it remains associated with elevated risks of preterm birth and potential nicotine-mediated neurodevelopmental disruptions. Dual use presents the highest risk profile, compounding exposure to both combustion products and concentrated nicotine aerosols.

A major methodological limitation in current human literature remains residual confounding attributable to unmeasured socio-demographic factors, baseline smoking history, incomplete reporting of exposure timing/trimester, and self-reported measurement bias. Future research must prioritize rigorous, prospective longitudinal studies that incorporate validated biomarkers of exposure (e.g., urine cotinine, NNAL, heavy metals) and standardized device and e-liquid tracking. In the clinical setting, healthcare providers counseling pregnant adolescents must provide clear, evidence-based guidance emphasizing that ECs are neither harmless nor FDA-approved smoking cessation aids during pregnancy.

## Data Availability

The original contributions presented in the study are included in the article/supplementary material, further inquiries can be directed to the corresponding author.
